# Surveillance for respiratory syncytial virus and parainfluenza virus among patients hospitalized with pneumonia in Sarawak, Malaysia

**DOI:** 10.1371/journal.pone.0202147

**Published:** 2018-08-15

**Authors:** Jane K. Fieldhouse, Teck-Hock Toh, Wei-Honn Lim, Jakie Ting, Siaw-Jing Ha, King-Ching Hii, Cheng-Ing Kong, Toh-Mee Wong, See-Chang Wong, Tyler E. Warkentien, Gregory C. Gray

**Affiliations:** 1 Duke Global Health Institute, Duke University, Durham, North Carolina, United States of America; 2 Division of Infectious Diseases, Duke University School of Medicine, Durham, North Carolina, United States of America; 3 Clinical Research Center, Sibu Hospital, Sibu, Sarawak, Malaysia; 4 Department of Paediatrics, Sibu Hospital, Sibu, Sarawak, Malaysia; 5 Faculty of Medicine, SEGi University Sibu Clinical Campus, Sibu Hospital, Sibu, Sarawak, Malaysia; 6 Kapit Hospital, Kapit, Sarawak, Malaysia; 7 Department of Medicine, Sibu Hospital, Sibu, Sarawak, Malaysia; 8 United States Naval Medical Research Center-Asia, Singapore, Singapore; 9 Emerging Infectious Diseases Program, Duke-NUS Medical School, Singapore, Singapore; Kliniken der Stadt Köln gGmbH, GERMANY

## Abstract

**Background:**

Respiratory syncytial virus (RSV) and parainfluenza virus (PIV) are frequent causes of pneumonia and death among children at Sibu and Kapit Hospitals in Sarawak, Malaysia.

**Objectives:**

To determine the prevalence and risk factors for RSV subtypes A and B and PIV types 1–4 among patients hospitalized with pneumonia.

**Methods:**

In a cross-sectional, pilot study nasopharyngeal swabs were studied with real-time reverse transcription polymerase chain reaction assays. Concurrently, we helped Sibu and Kapit Hospitals adapt their first molecular diagnostics for RSV and PIV.

**Results:**

Of 129 specimens collected (June to July 2017), 39 tested positive for RSV-A (30.2%), two were positive for RSV B (1.6%), one was positive for PIV-3 (0.8%) and one was positive for PIV-4 (0.8%). No samples were positive for PIV-1 or PIV-2. Of the 39 RSV-A positive specimens, 46.2% were collected from children under one year of age and only 5.1% were from patients over the age of 18. A multivariable analysis found the odds of children <1 year of age testing positive for RSV-A were 32.7 (95% CI: 3.9, 276.2) times larger than >18 years of age, and the odds of patients hospitalized at Kapit Hospital testing positive for RSV-A were 3.2 (95% CI: 1.3, 7.8) times larger than patients hospitalized at Sibu Hospital.

**Conclusion:**

This study found an unusually high prevalence of RSV-A among pneumonia patients admitted to the two hospitals. Subsequently, Sibu Hospital adapted the molecular assays with the goal of providing more directed care for such pneumonia patients.

## Background

In 2015, an estimated 2.7 million deaths worldwide (all ages) were attributable to lower respiratory infections [1]. While international surveillance for influenza viruses is routinely conducted for the purposes of developing seasonal vaccines, surveillance for non-influenza respiratory viruses, including respiratory syncytial virus (RSV), and parainfluenza virus (PIV), is less well established [2]. Recent publications have described these gaps in surveillance, recognizing the barrier of timely diagnostic testing and the need for the increased use of multiplexing molecular assays to enhance the sensitivity of respiratory virus detection [2, 3].

RSV, a single-stranded negative-sense RNA virus in the genus *Pneumovirus* in the Paramyxoviridae family has two antigenic subgroups, A and B, which can co-circulate but typically differ by season and region [4]. Estimates of the overall mortality among children due to RSV in 2015 range from 36,363 to 118,200 deaths globally [5, 6]. RSV has also been found to have a disease burden similar to that of non-pandemic Influenza A within elderly adult populations [7]. Worldwide, there are currently more than 50 RSV vaccine trials and nearly as many antiviral drug trials for RSV infection being evaluated in various phases of clinical trials [2, 3, 8–10]. Immune prophylaxis with targeted monoclonal antibodies (mAbs) are now available for high-risk infants and children [11].

Parainfluenza viruses are single-stranded, negative-sense RNA viruses in the Paramyxoviridae family. There are four primary types of human PIVs, PIV1-4. While PIV-3 infection is most prevalent, all four types of PIVs cause pneumonia, particularly among children and infants. Today there are 14 active vaccine trials for human PIV-3 [2].

A meta-analysis of the global burden of childhood pneumonia found the greatest proportion of severe pneumonia cases occurred in South East Asia, where 5.44 million out of 14.11 million severe cases worldwide occurred [12]. Sibu Hospital in Sarawak, Malaysia, has experienced increasing pneumonia admissions in recent years, with 1,611 admissions in 2013, 1,607 admissions in 2014, and 1,903 admissions in 2015. Sibu Hospital is a referral hospital for central Sarawak, with a bed capacity of 730, serving six other smaller district hospitals and a population of more than 725,400 in the central region of Borneo Island. A nearby district hospital, Kapit Hospital, while smaller, also sees a relatively large number of pneumonia admissions (approximately 300) per year considering the small rural population of 130,800 it serves. Kapit is located 120 kilometers south-east of Sibu, currently accessible only by boat. Kapit Hospital is a government hospital with 134 beds that serves as the referral hospital for several smaller towns along the Rajang River [13]. Until this study was conducted, the hospitals essentially have no onsite viral diagnostic capabilities.

## Objectives

In this six-week, cross-sectional pilot study we sought to estimate prevalence and to identify risk factors for RSV and PIV molecular detections among pediatric and adult pneumonia admissions at Sibu and Kapit Hospitals.

## Methods

This student project was a sub-investigation of an ongoing pneumonia etiology study conducted in in the same hospitals. Inclusion and exclusion enrollment criteria were adapted from two United States, large and comprehensive, community-based pneumonia studies (supporting information S1 Table). [14, 15]. All patients of more than 30 days of age admitted to Sibu or Kapit Hospitals were recruited after medical officers (MOs) assessed them for evidence of an acute respiratory tract infection consistent with pneumonia assessed by chest radiography, read by MOs, within 72 hours of hospitalization [14].

MOs from both hospitals were trained in enrollment procedures. Due to the dependency upon MOs for enrollment, this study used convenience sampling. Written informed consent was obtained from enrolled adults over 18 years of age and from parents or guardians of children under 7 years of age. Assent was obtained for those children enrolled between 7 and 18 years of age, along with written informed consent from their parents or legal guardians. These procedures were approved by the Medical Research and Ethics Committee, Ministry of Health, Malaysia, the Duke University Health System Institutional Review Board and the Naval Medical Research Center Asia Human Research Protection Program (HRPO # W911QY-16-D-0058).

MOs administered a questionnaire collecting demographic and risk factor data then collected and placed one nasopharyngeal swab into 3 mL of sterile viral transport medium. All specimens were stored at -80°C until RNA extraction was performed using QIAmp Cador Pathogen Mini Kit (cat. 54106). Extractions (100 μL) were then stored in cryogenic vials in a -80°C freezer until ready for real-time reverse transcription polymerase chain reaction (rRT-PCR). Primers and probes were identified and validated following a literature review of van de Pol et al. (2007) [16]. Cycling conditions for the six singleplex assays were identical (supporting information S2 Table). rRT-PCR was conducted on a BioRad CFx96 C1000 Touch Thermal Cycler Real-Time system. Cycle threshold (Ct) value cut-offs were determined based on a literature review: Ct values <38 were positive; Ct values 38 to 40 were considered suspect; and Ct values >40 were considered negative [16–19]. A positive control and a no-template control were included in each run.

Questionnaire data and rRT-PCR results were entered into REDCap version 7.0 and data were imported into STATA version 15.0 (StataCorp, College Station, TX). To assess for potential risk factors, an initial bivariate screening was conducted to assess the association between covariates of interest and the outcome of positive viral detection by rRT-PCR. Logistic regression was used to assess the following covariates as possible risk factors: age category, gender, location (hospital), week of enrollment, ethnicity, pre-existing medical conditions, current medications, and history of animal exposure. Pearson’s chi-square test was used for categorical variables. Those predictors with a bivariate test statistic *p*-value ≤0.1 were included in a stepwise, backward elimination logistic regression model. Predictors with a *p*-value <0.05 were retained in a final model to calculate adjusted odds ratios (OR) and 95% confidence interval (CI).

## Results

From June 15 to July 27, 2017, we enrolled 129 patients who met the eligibility criteria out of a total 567 patients who were hospitalized with pneumonia at Sibu and Kapit Hospitals (supporting information S3 Table). For the purpose of this analysis, three suspect-positive RSV-A samples, which were run a second time for validation and revealed consistent amplification curves, were considered positive. Thus, 39 samples were positive for RSV-A (30.2%), two were positive for RSV-B (1.6%), one was positive for PIV-3 (0.8%) and one was positive for PIV-4 (0.8%). No samples tested positive for PIV-1 or PIV-2. Due to low prevalence, RSV-B and PIV1-4 outcomes were not included in risk factor modeling.

A bivariate analysis of dichotomous risk factors against the outcome of RSV-A positive found potentially important risk factors to include: age category, location, several pre-existing conditions and several medications. The final stepwise, backward elimination logistic regression model included age and location variables. When controlling for age, patients enrolled at Kapit Hospital had a higher adjusted OR of testing positive for RSV-A (adjusted OR = 3.2, 95% CI: 1.3, 7.8) (Table 1). When controlling for location, the adjusted OR of RSV-A infection for participants <1 year was 32.7 (95% CI: 3.9, 276.2), and the adjusted OR of RSV-A infection for participants ages 1 to 5 years was 14.9 (95% CI: 1.5, 100.8).

**Table 1:** Significant risk factors for molecular detection of RSV-A.

Risk Factor	Total Number	RSVA + (%)	RSVA—(%)	Unadjusted OR (95% CI)	Adjusted OR (95% CI)
Hospital					
*Kapit*	50	23 (46.0)	27 (54.0)	3.4 (1.5, 7.3)	3.2 (1.3, 7.8)
*Sibu*	79	16 (20.3)	63 (79.7)	Ref.	Ref.
Age Category ^a^ (years)					
*<1*	32	18 (56.2)	14 (43.8)	37.3 (4.5, 308.2)	32.7 (3.9, 276.2)
*1–5*	53	18 (34.0)	35 (66.0)	14.9 (1.9, 118.5)	12.4 (1.5, 100.8)
*6–17*	10	1 (10.0)	9 (90.0)	3.2 (0.2, 56.9)	2.5 (0.1, 46.1)
*>18*	30	1 (3.3)	29 (96.7)	Ref.	Ref.

OR: Odds Ratio; RSV: Respiratory syncytial virus

^a^ Date of birth data missing for one RSV-A positive patient

## Discussion

The study found a remarkably high prevalence of RSV-A (30.2% overall) among patients with pneumonia admitted to the two hospitals. Given previous studies of respiratory viruses in Malaysia, we expected to see an RSV prevalence between 15 to 20% [20, 21]. The prevalence was particularly high at Kapit Hospital (46.0%). The detection of RSV A is in keeping with the finding that globally, RSV group A tends to be more prevalent than RSV group B [22]. We found that RSV subtype A led to more hospitalizations than RSV B; however, there are mixed findings as to the clinical significance of the different genotype infections [6, 22, 23].

Based on RSV studies conducted between 1982 and 2008, the RSV season in Malaysia begins in July, peaks between September and December, and ends in March for a duration of about 36 weeks [24]. Our findings demonstrate RSV season may begin earlier than July, though data from an ongoing pneumonia study will provide a snapshot of year-long data on the seasonality of RSV in Sarawak, in addition to influenza viruses, adenoviruses, coronaviruses and enteroviruses. Because data collection occurred in June and July, the two months following the Gawai celebration in Sarawak, it is possible that community gatherings might have contributed to the increased prevalence of RSV infection. This would be especially true in Kapit, which is a smaller and more isolated town that attracts nonresident travelers during the Gawai celebration. The population of Kapit is largely made up of ethnic groups that reside in traditional or modern longhouses, with rows of private single-family units running perpendicular to a hall of public, communal space. Of the RSV-A patients in our study, 60% reported residing in households with six or more cohabitants; however, while the questionnaire captured household size, it failed to capture household type (longhouse versus single-family home).

Though ethnicity was not found to be a statistically significant risk factor for RSV-A infection, 26 of the RSV-A positive patients were of Iban ethnicity (66.7%). As of 2010, approximately 28% of the population in Sibu was Iban and 82% of the population in Kapit was Iban; in this study, 58% of patients in Sibu were Iban and 88% of patients in Kapit were Iban. Among those patients hospitalized in Kapit, a remaining 4% were Malay and 8% were indigenous local populations, including the nomadic hunter-gatherer Penan people.

This study was limited in its inference as we used convenience sampling and only sampled during June and July; we cannot exclude the possibility that our patients were the most seriously ill and that the prevalence of RSV and PIV will differ by seasons.

Strengths of the study include the use of singleplex rRT-PCR to sub-type RSV and PIV. While rRT-PCR is highly sensitive, it is possible that some infections could have been missed.

This study provides baseline surveillance data to estimate the prevalence of both RSV and PIV within the Sibu and Kapit pneumonia patient populations, which may help inform medical practitioners as to the etiology of pneumonia in Sarawak and guide future interventions and vaccine or pharmaceutical trials. With new treatments for both viruses on the horizon, continued studies are required to assess RSV and PIV trends in Sarawak.

## Supporting information

S1 Table Inclusion and exclusion criteria by age. Adapted from: Jain, S., et al., Community-acquired pneumonia requiring hospitalization among U.S. adults. *New England Journal of Medicine*, 2015. 373(5): 415–427 and Jain, S., et al., Community-acquired pneumonia requiring hospitalization among U.S. children. *New England Journal of Medicine*, 2015. 372(9): 835–845.
(DOCX)

S2 Table Cycling conditions of RSV and PIV assays. (DOCX)

S3 Table Characteristics of enrolled subjects. COPD: Chronic obstructive pulmonary disease; § dyslipidemia, gout, obstructive sleep apnea, hyperthyroidism, benign prostatic hyperplasia, reactive airway disease, bronchiolitis obliterans, iron deficiency anemia; ' Penan, Ulu-Sekapan, Kadazan, Bisaya, Bugis, Dayak, Kenya; ‡ colchicine, allopurinol, aspirin, gout medication, multi-vitamin, iron supplement, Nafarin-A; † cats, dogs.
(DOCX)
