# Supplementary material for: Surveillance for respiratory syncytial virus and parainfluenza virus among patients hospitalized with pneumonia in Sarawak, Malaysia
Source: PLoS One. 2018 Aug 15;13(8):e0202147. doi: 10.1371/journal.pone.0202147 (PMC6093684; doi:10.1371/journal.pone.0202147)
Supplement: S1 Table — Adapted from: Jain, S., et al., Community-acquired pneumonia requiring hospitalization among U.S. adults. New England Journal of Medicine, 2015. 373(5): 415–427 and Jain, S., et al., Community-acquired pneumonia requiring hospitalization among U.S. children. New England Journal of Medicine, 2015. 372(9): 835–845. (DOCX) [file pone.0202147.s001.docx]

**S1 Table. Inclusion and exclusion criteria by age.**

| Inclusion Criteria Children (1 month to 18 years) | Inclusion Criteria Adults (18 years or more) |
| --- | --- |
| - They were admitted to Sibu or Kapit hospital; - Have evidence of acute infection, defined as reported fever or chills, documented fever or hypothermia, or leukocytosis or leukopenia; - Have evidence of an acute respiratory illness, defined as new cough or sputum production, chest pain, dyspnea, tachypnea, abnormal lung examination, or respiratory failure; - A parent or legal guardian provides written informed consent. In addition to parental consent, signed assent document will be sought from children 7 to 18 years of age. - The evidence of illness is consistent with pneumonia as assessed by means of chest radiography within 72 hours before or after admission. | - They were admitted to Sibu or Kapit hospital on the basis of a clinical assessment by the treating clinician; - Have evidence of acute infection, defined as reported fever or chills, documented fever or hypothermia, leukocytosis or leukopenia, or new altered mental status; - Have evidence of an acute respiratory illness, defined as new cough or sputum production, chest pain, dyspnea, tachypnea, abnormal lung examination, or respiratory failure; - Have evidence consistent with pneumonia as assessed by means of chest radiography by the clinical team within 48 hours before or after admission. |
| Exclusion Criteria Children (1 month to 18 years) | Exclusion Criteria Adults (18 years or more) |
| - If they had been hospitalized recently (<7 days for immunocompetent children and <90 days for immunosuppressed children) - If they had already been enrolled in this study within the previous 28 days - If they resided in an extended-care facility - If they had an alternative diagnosis of a respiratory disorder - If they were newborns who never left the hospital - If they had a tracheostomy tube - If they had cystic fibrosis or - If they had cancer with neutropenia - If they had received a solid-organ or hematopoietic stem-cell transplant within the previous 90 days - If they had active graft-versus-host disease or bronchiolitis obliterans - If they had human immunodeficiency virus infection with a CD4 cell count of less than 200 per cubic millimeter (or a percentage of CD4 cells <14%). | - If they had been hospitalized recently (<28 days for immunocompetent patients and <90 days for immunosuppressed patients) - If they had already been enrolled in this study within the previous 28 days - If they were functionally dependent nursing home residents - If they had a clear alternative diagnosis - If they had undergone tracheotomy - If they had a percutaneous endoscopic gastrostomy tube - If they had cystic fibrosis - If they had cancer with neutropenia - If they had received a solid-organ or hematopoietic stem-cell transplant within the previous 90 days - If they had active graft-versus-host disease - If they had bronchiolitis obliterans - If they had human immunodeficiency virus infection with a CD4 cell count of less than 200 per cubic millimeter. |

Adapted from: Jain, S., et al., Community-acquired pneumonia requiring hospitalization among U.S. adults. *New England Journal of Medicine*, 2015. 373(5): 415-427 and Jain, S., et al., Community-acquired pneumonia requiring hospitalization among U.S. children. *New England Journal of Medicine*, 2015. 372(9): 835-845.

**SUPPLEMENTAL REFERENCES:**

1. Jain S, Self WH, Wunderink RG, Fakhran S, Balk R, Bramley AM, et al. Community-Acquired Pneumonia Requiring Hospitalization among U.S. Adults. New England Journal of Medicine. 2015;373(5):415-27.

2. van de Pol AC, van Loon AM, Wolfs TFW, Jansen NJG, Nijhuis M, Breteler EK, et al. Increased Detection of Respiratory Syncytial Virus, Influenza Viruses, Parainfluenza Viruses, and Adenoviruses with Real-Time PCR in Samples from Patients with Respiratory Symptoms. Journal of Clinical Microbiology. 2007;45(7):2260-2.
