# Supplementary material for: Surveillance for respiratory syncytial virus and parainfluenza virus among patients hospitalized with pneumonia in Sarawak, Malaysia
Source: PLoS One. 2018 Aug 15;13(8):e0202147. doi: 10.1371/journal.pone.0202147 (PMC6093684; doi:10.1371/journal.pone.0202147)
Supplement: S2 Table — (DOCX) [file pone.0202147.s002.docx]

**S2 Table. Cycling conditions of RSV and PIV assays.**

| **Step** | **Temp** | **Time** | **#** |
| --- | --- | --- | --- |
| 1 | 50°C | 30 min | x1 |
| 2 | 95°C | 2 min | x1 |
| 3 | 95°C | 15 sec |  |
|  | 60°C | 30 sec | X40 |
