# Supplementary material for: Surveillance for respiratory syncytial virus and parainfluenza virus among patients hospitalized with pneumonia in Sarawak, Malaysia
Source: PLoS One. 2018 Aug 15;13(8):e0202147. doi: 10.1371/journal.pone.0202147 (PMC6093684; doi:10.1371/journal.pone.0202147)
Supplement: S3 Table — COPD: Chronic obstructive pulmonary disease; § dyslipidemia, gout, obstructive sleep apnea, hyperthyroidism, benign prostatic hyperplasia, reactive airway disease, bronchiolitis obliterans, iron deficiency anemia; ' Penan, Ulu-Sekapan, Kadazan, Bisaya, Bugis, Dayak, Kenya; ‡ colchicine, allopurinol, aspirin, gout medication, multi-vitamin, iron supplement, Nafarin-A; † cats, dogs. (DOCX) [file pone.0202147.s003.docx]

**S3 Table. Characteristics of enrolled subjects.**

| **Characteristics** | **All Total Number (%)** | **Sibu Number (%)** | **Kapit Number (%)** |
| --- | --- | --- | --- |
| Total | 129 (100) | 79 (61.2) | 50 (38.8) |
| Sex |  |  |  |
| Female | 56 (43.4) | 36 (45.6) | 20 (40.0) |
| Male | 73 (56.6) | 43 (54.4) | 30 (60.0) |
| Age groups |  |  |  |
| <1 | 32 (24.8) | 18 (22.8) | 14 (28.0) |
| 1-5 | 53 (41.1) | 31 (39.2) | 22 (44.0) |
| 6-18 | 10 (7.8) | 6 (7.6) | 4 (08.0) |
| >18 | 30 (23.3) | 24 (30.4) | 6 (12.0) |
| missing | 4 (3.1) | 0 (0.0) | 4 (8.0) |
| Household size |  |  |  |
| ≤2 | 16 (12.4) | 9 (11.4) | 7 (14.0) |
| 3-5 | 48 (37.2) | 29 (36.7) | 19 (38.0) |
| 6-10 | 52 (40.3) | 30 (38.0) | 22 (44.0) |
| >10 | 13 (10.1) | 11 (13.9) | 2 (4.0) |
| Ethnicity |  |  |  |
| Iban | 90 (69.8) | 46 (58.2) | 44 (88.0) |
| Malay | 10 (7.8) | 8 (10.1) | 2 (4.0) |
| Chinese | 7 (5.4) | 7 (8.9) | 0 (0.0) |
| Bidayuh | 1 (0.8) | 0 (0.0) | 1 (2.0) |
| Melanau | 13 (10.1) | 13 (16.5) | 0 (0.0) |
| Other' | 8 (6.2) | 5 (6.3) | 3 (6.0) |
| Pre-existing conditions |  |  |  |
| Hypertension | 15 (11.6) | 11 (23.9) | 4 (8.0) |
| Diabetes mellitus | 5 (3.9) | 3 (03.8) | 2 (4.0) |
| Heart disease | 5 (3.9) | 5 (6.3) | 0 (0.0) |
| COPD | 10 (7.8) | 9 (11.4) | 1 (02.0) |
| Asthma | 5 (3.9) | 4 (5.1) | 1 (02.0) |
| Cancer | 1 (0.8) | 1 (1.3) | 0 (0.0) |
| Other§ | 17 (13.2) | 16 (20.3) | 1 (2.0) |
| None | 92 (71.3) | 48 (60.8) | 44 (88.0) |
| Medical treatment in last 6 months |  |  |  |
| Diabetes medicine | 5 (3.9) | 3 (3.8) | 2 (4.0) |
| Hypertension medicine | 14 (10.9) | 11 (13.9) | 3 (6.0) |
| Oral corticosteroid | 1 (0.8) | 1 (1.3) | 0 (0.0) |
| Inhaled corticosteroid | 19 (14.7) | 16 (20.3) | 3 (6.0) |
| Cancer medicine | 0 (0.0) | 0 (0.0) | 0 (0.0) |
| Others‡ | 7 (5.4) | 7 (8.9) | 0 (0.0) |
| None | 98 (76.0) | 53 (67.1) | 45 (90.0) |
| Animals touched or within 1 meter in the last 30 days |  |  |  |
| Pigs | 8 (6.2) | 6 (7.6) | 2 (4.0) |
| Chickens | 26 (20.2) | 13 (16.6) | 13 (26.0) |
| Ducks | 9 (7.0) | 5 (6.3) | 4 (8.0) |
| Other poultry | 4 (3.1) | 4 (5.1) | 0 (0.0) |
| Horses | 0 (0.0) | 0 (0.0) | 0 (0.0) |
| Cows | 1 (0.8) | 1 (1.3) | 0 (0.0) |
| Goats | 0 (0.0) | 0 (0.0) | 0 (0.0) |
| Other† | 47 (36.4) | 34 (43.0) | 13 (26.0) |

COPD: Chronic obstructive pulmonary disease; § dyslipidemia, gout, obstructive sleep apnea, hyperthyroidism, benign prostatic hyperplasia, reactive airway disease, bronchiolitis obliterans, iron deficiency anemia; ' Penan, Ulu-Sekapan, Kadazan, Bisaya, Bugis, Dayak, Kenya; ‡ colchicine, allopurinol, aspirin, gout medication, multi-vitamin, iron supplement, Nafarin-A; † cats, dogs
